# Advanced Urea Precursors Driven NiCo_2_O_4_ Nanostructures Based Non-Enzymatic Urea Sensor for Milk and Urine Real Sample Applications

**DOI:** 10.3390/bios13040444

**Published:** 2023-03-31

**Authors:** Sanjha Mangrio, Aneela Tahira, Abdul Sattar Chang, Ihsan Ali Mahar, Mehnaz Markhand, Aqeel Ahmed Shah, Shymaa S. Medany, Ayman Nafady, Elmuez A. Dawi, Lama M. A. Saleem, E. M. Mustafa, Brigitte Vigolo, Zafar Hussain Ibupoto

**Affiliations:** 1 Dr. M.A. Kazi Institute of Chemistry, University of Sindh, Jamshoro 76080, Pakistanasattar.chang@usindh.edu.pk (A.S.C.);; 2 Institute of Chemistry, Shah Abdul Latif University, Khairpur Mirs 66111, Pakistan; 3 Department of metalluargy and Materials, NED University of Engineering and Technology, Karachi 75270, Pakistan; 4 Department of Chemistry, Faculty of Science, Cairo University, Giza 12613, Egypt; 5 Department of Chemistry, College of Science, King Saud University, Riyadh 11451, Saudi Arabia; 6 Nonlinear Dynamics Research Centre (NDRC), Ajman University, Ajman P.O. Box 346, United Arab Emirates; 7 Biomolecular Science, Earth and Life Science, Amsterdam University, 1081 HV Amsterdam, The Netherlands; 8 Department of Sciences and Technology, Linköping University, SE-601 74 Norrköping, Sweden; 9 The Institut Jean Lamour (IJL), Université de Lorraine, CNRS, F-54000 Nancy, France

**Keywords:** urea precursors, NiCo_2_O_4_ nanostructures, non-enzymatic sensor, alkaline conditions

## Abstract

The electrochemical performance of NiCo_2_O_4_ with urea precursors was evaluated in order to develop a non-enzymatic urea sensor. In this study, NiCo_2_O_4_ nanostructures were synthesized hydrothermally at different concentrations of urea and characterized using scanning electron microscopy and X-ray diffraction. Nanostructures of NiCo_2_O_4_ exhibit a nanorod-like morphology and a cubic phase crystal structure. Urea can be detected with high sensitivity through NiCo_2_O_4_ nanostructures driven by urea precursors under alkaline conditions. A low limit of detection of 0.05 and an analytical range of 0.1 mM to 10 mM urea are provided. The concentration of 006 mM was determined by cyclic voltammetry. Chronoamperometry was used to determine the linear range in the range of 0.1 mM to 8 mM. Several analytical parameters were assessed, including selectivity, stability, and repeatability. NiCo_2_O_4_ nanostructures can also be used to detect urea in various biological samples in a practical manner.

## 1. Introduction

Urea is referred to as carbamide and carbonyl diamide and is produced in the human body in an amount ranging from 7–80% through the metabolism of nitrogenous compounds. In clinical terms, it is considered to be extremely important due to its presence in urine and blood. In addition to being present in the human body, urea is also commonly found in fertilizers, agriculture, dairy industries, and food products. Many developing countries use urea as an additive in dairy products, causing conditions such as ulcers, acidity, ulcers, kidney disease, and indigestion. Generally, urea in milk may be present at concentrations of 18–40 mg per deciliter [1]. As a result, the quantification of milk urea nitrogen will allow more accurate control of dairy cattle’s diet to avoid issues related to reproduction and health [2]. Consequently, it is apparent that a selective and sensitive monitoring method must be developed in order to quantify urea. The urea present in the human body flows through the bloodstreams to the kidneys, where it is eventually excreted as urine [3], the result of final protein metabolism [4]. In recent years, non-enzymatic sensing approaches have gained popularity among researchers due to their unique characteristics, such as selectivity, stability, and low cost. For this to be achieved, functional materials must be designed in such a way that they are capable of detecting analytes under the required physiological conditions. Currently, non-enzymatic urea sensing methods are limited, making it imperative to synthesize new materials that have modified properties. Different analytical methods have been used to determine urea, including colorimetry [5], infrared spectroscopy [6], fluorimetry [7], gas chromatography (GC) [8], high performance liquid chromatography (HPLC) [9], liquid chromatography-mass spectrometry (LCMS) [10], electroanalytical [11], and chemiluminescence [12]. Generally, the oxidation potential of uric acid and urea could be found in the range of 0.3–0.35 V and 0.5–0.6 V, respectively, as reported elsewhere. However, some variations with the oxidation potential of uric acid and urea can be observed, depending on the nature of the catalytic material. It is important to note that even though these methods are well established, they are extremely time consuming, extremely expensive, and require skilled personnel for use. For many of these methods, urease enzyme is utilized for the catalytic conversion of urea into ammonia and carbon dioxide; however, the major problem is the stability and storage of the enzyme. Urease enzyme has been investigated to develop enzyme-free sensors using a range of materials including cobalt oxide [13,14], tin oxide [15], nickel cobalt oxide [16], nickel/cobalt oxide graphene composite [17], and polypyrrole/platinum composite [18]. Enzyme-free sensing of urea has certain limitations, including poor catalytic properties, low conductivity, and high charge transfer resistance. This necessitates research on the development of new urea sensors. Among these materials, bimetallic oxides have a better electrical conductivity and lower charge transfer resistance than single metal oxides. NiCo_2_O_4_ is a nickel-cobalt oxide (bi-metallic oxide) that has excellent properties for driving the urea reaction with significant reaction kinetics [19]. NiCo_2_O_4_ nanostructures have been prepared by numerous methods, including nano-casting [20], hydroxide decomposition [21], electrodeposition [22], coprecipitation [23], and solvothermal synthesis [24]. As a result of their high complexity and energy consumption, these methods are not suitable for the development of enzyme-free sensors for industrial purposes. It is, therefore, urgent to design and propose a rapid method that has several eco-friendly feathures, is environmentally friendly, low cost, and can be scaled up for the growth of nanostructures that outperform NiCo_2_O_4_. Wet chemical methods have gained significant attention in recent years due to their fast, facile, and promising characteristics. These methods are able to control the dimensions and shape of nanostructured materials by controlling the reaction conditions, such as pH, temperature, precursor concentration, and solvent volume [24,25,26,27,28]. Previous studies have employed hydrothermal methods to investigate the role of urea concentration on NiCo_2_O_4_ nanostructures for electrochemical supercapacitor applications [25]. Our previous work used NiCo_2_O_4_ nanoneedles to develop a non-enzymatic urea sensor, and the concentrations of urea and cobalt chloride salt were 3.3 M and 0.1 M, respectively [16]. In a previous study [16], we primarily used 0.1 M cobalt chloride and 3.3 M urea concentrations for the synthesis of NiCo_2_O_4_ and evaluated non-enzymatic urea detection by cyclic voltammetry. However, later in this study, we realized that urea could be sensed successfully if the urea concentrations were varied and evaluated for their effects on NiCo_2_O_4_ nanostructures and crystal quality in order to develop highly sensitive nonenzymatic urea sensors. The varying concentration of urea can also provide an insight into the reducing properties of nanostructured materials. During the sensing of urea through a wide linear range with a low detection limit, a highly sensitive electrical signal was generated during the sensing process. Our previous study did not highlight the role of urea both as a reducing agent and as a source of hydroxyl ions for the binding of metallic ions to form metal hydroxides. This fundamental understanding of precursor’s role in tuning surface properties may lead to the development of new generation electrochemical sensors.

This study was primarily motivated by the goal of tailoring the properties of NiCo_2_O_4_ nanostructures under the influence of urea concentrations. As a hydroxyl source, urea binds with metallic ions like nickel and cobalt to form a hydroxide phase. Furthermore, urea can function as a reducing agent, so different concentrations of urea can affect the surface properties of NiCo_2_O_4_ nanostructures. A previous study has indicated that NiCo_2_O_4_ nanostructures are synthesized using hydroxyl source from urea. However, the reducing agent properties of urea have not been studied for the purpose of surface modification, as well as tailoring the surface properties for the purpose of developing non-enzymatic urea sensors. Furthermore, the present study provides a simple approach to enhance the electrochemical activity of NiCo_2_O_4_ nanostructures prepared with three different urea concentrations. This approach is without the use of any other additive during the synthesis of material. Taking into account the high concentration of urea used during the synthesis of NiCo_2_O_4_ nanostructures, we believe that variation in urea concentration can tune the functionality of the material into a linear range of urea detection by developing a non-enzymatic urea sensing configuration. Even so, NiCo_2_O_4_ still needs to be improved in terms of shape, crystalline properties, catalytic activities, and conductivity so as to facilitate the superfast interface phenomenon during the enzyme-free approach to the sensing of small biomolecules, such as urea. Toward the development of non-enzymatic urea sensors for practical applications, we investigated the optimized role of urea concentration during the chemical reaction on the enhanced performance of NiCo_2_O_4_ nanostructures.

Our study demonstrates the method of hydrothermal synthesis of different NiCo_2_O_4_ nanostructures by varying the concentration of urea during the process. An SEM and an XRD were used to determine the morphology and crystallization of the samples. Using an optimized urea concentration for growing NiCo_2_O_4_ nanostructures, an advanced non-enzymatic urea sensor configuration has been successfully developed, and a wide linear range has been obtained for the detection of urea from 0.1 mM to 8 mM by a chronoamperometric electrochemical method.

## 2. Materials and Methods Used

### 2.1. Chemical Reagents

Several chemicals have been purchased from Sigma Aldrich Karachi Pakistan, including cobalt chloride hexahydrate (98%), nickel chloride hexahydrate (99.9%), urea (99%), ethanol (99%), potassium dihydrogen phosphate (98%), disodium hydrogen phosphate (98%), sodium chloride (99.5%), and sodium hydroxide (97%). All chemicals received are of high analytical quality and can be applied without further treatment. A deionized water solution was used to prepare growth precursors for the synthesis of the materials.

### 2.2. Effect of Various Urea Concentrations on the Structure Orientation of NiCo_2_O_4_ Nanostructures during Hydrothermal Method

**Scheme 1:**
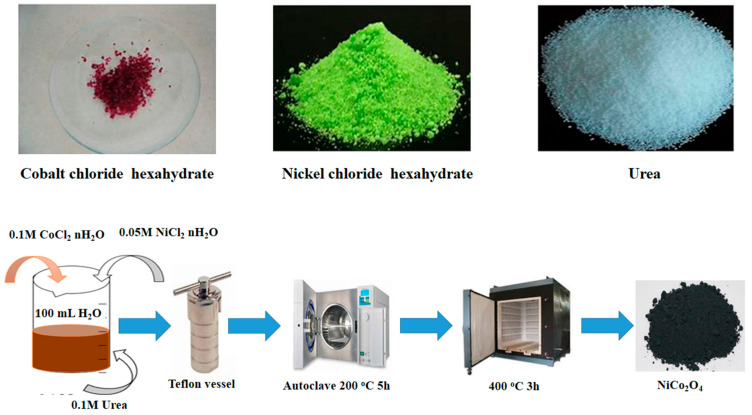
Synthesis of NiCo_2_O_4_ nanostructured materials by hydrothermal method followed by calcination in air.

In this study, bimetallic oxide nanowires of NiCo_2_O_4_ were prepared by a hydrothermal method in the presence of various urea concentrations, 0.05 M, 0.075 M, and 0.1 M, keeping the constant 0.1 M cobalt chloride hexahydrate and 0.015 M nickel chloride hexahydrate concentrations. The main purpose of this study was to highlight the importance of the influence of urea on the structure and electrochemical properties of NiCo_2_O_4_ nanostructures towards non-enzymatic urea sensing applications. Three beakers of growth solution were prepared with the above mentioned urea concentrations, and they were placed in a Teflon vessel of 100 mL volume and sealed in a stainless steel autoclave. Then, the hydrothermal growth process was started at 120 °C for 5 h in an electric oven. Afterwards, the bimetallic hydroxide product prepared with different urea concentrations was collected, washed several times, and dried at room temperature overnight. The hydroxide phase was transformed into the bimetallic oxide phase at 400 °C for 4 h in air using a thermal annealing process. Finally, we obtained NiCo_2_O_4_ nanostructures, and they were used for the structural and urea sensing applications. NiCo_2_O_4_ nanostructures can be synthesized in a highly repeatable manner with almost the same electrochemical performance if the conditions reported in this paper are followed properly, thereby demonstrating the same urea sensing response when applied to practical environments. The synthesis process of NiCo_2_O_4_ nanostructures using various urea concentrations is also represented by following Scheme 1.

### 2.3. Crystal Quality and Morphology Investigations of Various NiCo_2_O_4_ Nanostructures

Morphological aspects of prepared NiCo_2_O_4_ nanostructures with three different urea concentrations were studied using scanning electron microscopy (SEM) with a ZEISS Gemini SEM 500 connected to field emission gun. The crystal quality of synthesized NiCo_2_O_4_ nanostructures was also studied by powder X-ray diffraction technique using Bruker D8 Advance diffractometer with source CuKα radiation of wavelength 1.54050 Å, employed at 45 mA and 45 kV. Electrochemical characterization was done with cyclic voltammetry (CV) in a potential window from −0.8 V to 8 V at 50 mV/s except the scan rate study, and chronoamperometry was performed at 0.6 V 300 s.

### 2.4. Different Urea Concentrations’ Role towards the Enhancement of Electrochemical Properties of NiCo_2_O_4_ Nanostructure for the Development of Advanced Non-Enzymatic Urea Sensors

A three-electrode cell set up was used to investigate the electrochemical properties of NiCo_2_O_4_ nanostructures prepared with different concentrations of urea during the growth process. Using a reference electrode of silver-silver chloride (Ag/AgCl, 3.0 M KCl), counter electrode of platinum sheet and working electrode of glassy carbon electrode (GCE) modified with different NiCo_2_O_4_ nanostructures were configured into an electrochemical cell set up. The GCE was cleaned with liquid alumina (0.3 M) slurry and silicon paper followed by washing with deionized water.

The modification of cleaned GCE was done with using 5 µL of well dispersion of 5 mg of NiCo_2_O_4_ nanostructures in 2 mL of deionized water and 0.5 mL of 5% Nafion polymeric binder. The drop cast method was used to modify the surface of GCE and used as a working electrode. Prior to electrochemical measurements, the 0.1 M electrolyte solution of NaOH was made and employed for the preparation of 0.1 M urea stock solution for the non-enzymatic sensing application. Different diluted solutions of urea analyte were prepared by dilution method when needed. The selectivity experiment was performed with different competing interfering agents during the urea sensing, and their concentrations of 0.1 mM were also prepared in 0.1 M NaOH. All experiments were performed at standard room temperature conditions. According to the reported works [29,30] with slight modifications, real milk samples were prepared for the practical application of the non-enzymatic urea sensor. The raw milk samples were purchased from a dairy shop located in the Jamshoro neighborhood. Initially, the raw milk samples were processed in order to remove any unwanted proteins and fat molecules. Approximately 10 mL of milk were poured into a centrifuge tube with 8 mL of acetic acid (*v*/*v*, 3%). A mechanical shaking process was then performed on the tube for 2 min. A centrifuge process was then conducted at 12,000 rpm for two min in order to remove the precipitated protein and fat- like products. Finally, the obtained milk liquid of 20 microliter was spiked with 0.M NaOH to quantify the urea from the milk samples.

## 3. Results and Discussion

### 3.1. Morphology and Crystalline Studies of as Prepared NiCo_2_O_4_ Nanostructures

**Figure 1:**
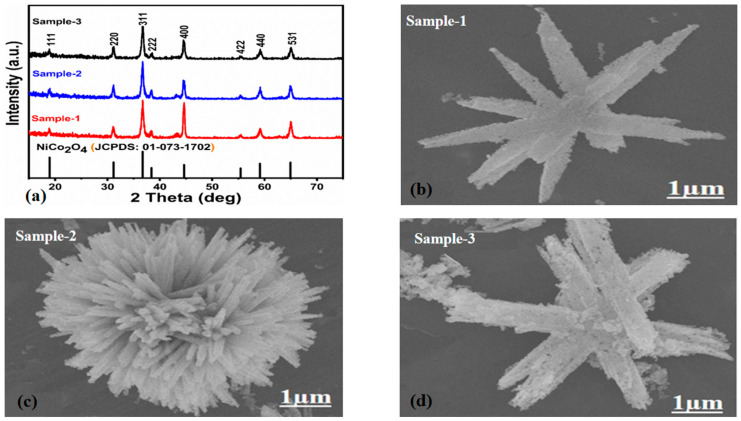
(**a**) XRD patterns of NiCo_2_O_4_ samples prepared with various concentrations of urea (Samples 1–3), (**b**–**d**) effect of various urea concentrations (Samples 1–3) on the morphology of NiCo_2_O_4_ nanomaterial.

As shown in Figure 1, NiCo_2_O_4_ nanostructures were characterized from a structural and a morphological perspective using three different urea concentrations. Three different urea concentrations can affect the crystal quality of NiCo_2_O_4_ nanostructures, as shown in Figure 1a, and three different urea concentrations can also affect their morphology, as shown in Figure 1b–d. We prepared three samples of NiCo_2_O_4_ nanostructures using different urea concentrations, such as 0.05 M, 0.075 M, and 0.1 M, and labelled them as Sample 1, Sample 2, and Sample 3. We investigated the effects of three different urea concentrations on the morphology and crystal quality of the NiCo_2_O_4_ nanostructures, which further contributed to the enhancement of electrochemical performance of enzyme-free urea sensing devices. Different urea concentrations were investigated with X-ray diffraction (XRD) to determine the effect on the crystal quality of NiCo_2_O_4_ nanostructures. The measured diffractograms are shown in Figure 1a. Crystal reflections were measured at two theta scanning angles of 10 to 80°, including (111), (220), (331), (222), (400), (422), (511), and (440), and it was demonstrated that NiCo_2_O_4_ nanostructures had the cubic crystal phase. The reference card 01-073-1704 supports the observed crystal phase information of NiCo_2_O_4_ nanostructures as prepared. XRD analysis revealed spinel structures in NiCo_2_O_4_ nanostructures, and there were no other diffraction patterns in the samples, indicating they were prepared using high-quality materials. According to the XRD study, the diffraction patterns at (400) were relatively more intense for Sample 1 than for Sample 3 and Sample 2 of NiCo_2_O_4_ nanostructures, suggesting that Sample 1 is preferred. NiCo_2_O_4_ nanostructures were also evaluated for their morphology in the presence of urea, and the results of typical SEM images are shown in Figure 1b–d. The urea concentration has shown significant influence on the morphology of NiCo_2_O_4_ nanostructures by sharpening the top surface of material and enhancing the texture of morphology by lowering the urea concentration, as can be seen from Figure 1b–d. According to SEM analysis, Samples 1 and 2 of NiCo_2_O_4_ nanostructures had sharp nanorod-like morphologies. In contrast, Sample 3 of NiCo_2_O_4_ nanostructures had platelet-like morphologies as shown in Figure 1b. An interesting finding is that different urea concentrations have a significant effect on NiCo_2_O_4_ nanostructure orientation, crystal quality, and morphology. Studies involving XRD and SEM suggest that such aspects of urea’s role in the structure of NiCo_2_O_4_ nanostructures may influence the final application of the material.

### 3.2. Non-Enzymatic Urea Sensor Based on NiCo_2_O_4_ Nanomaterial

**Figure 2:**
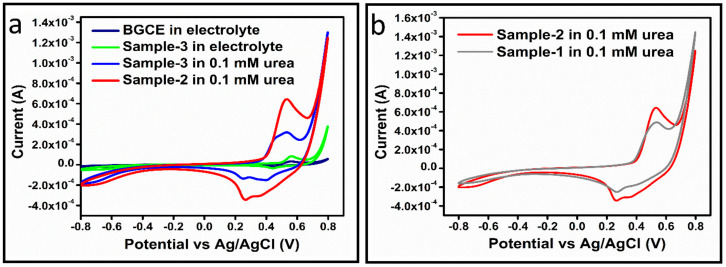
(**a**) CV curves of bare GCE (BGCE) and modified glassy carbon electrode (MGCE) with Sample 3 NiCo_2_O_4_ nanomaterial, Samples 1 and 2, 50 mV/s in the presence of 0.1 mM urea and only in 0.1 M NaOH, (**b**) CV curves of Samples 1 and 2 based NiCo_2_O_4_ nanomaterial, at 50 mV/s in the presence of 0.1 mM urea prepared in 0.1 M NaOH.

In Figure 2, cyclic voltammetry was used at a scan rate of 50 mV/s in 0.1 M NaOH as a supporting electrolyte. This was done in order to evaluate the electrochemical signal of NiCo_2_O_4_ nanomaterials for urea detection. This was accomplished by measuring the CV curves of pristine NiCo_2_O_4_ nanomaterial and Sample 1 with and without urea. As far as the electrolyte and analyte were concerned, the bare glassy carbon did not exhibit any redox behavior. While the pristine NiCo_2_O_4_ nanomaterial and Sample 1 have demonstrated weak redox properties in the electrolyte, when 0.1 mM urea concentrations were added to the electrolyte, the oxidation peak of Sample 1 became very prominent as compared to that of the pristine NiCo_2_O_4_ nanomaterial, indicating strong electrocatalytic properties displayed in Figure 2. NiCo_2_O_4_′s catalytic properties are influenced, in significant part, by the urea concentration in this CV study. This behavior of change of urea concentration during the growth of different NiCo_2_O_4_ nanomaterials has been studied for two samples of NiCo_2_O_4_ nanomaterials, as shown in Figure 2. In light of the decrease in the electrocatalytic properties of NiCo_2_O_4_ nanomaterials, a weak oxidation signal was observed as a result of the further reduction of urea concentration. In the CV analysis, it was determined that urea concentrations have a relatively noticeable effect on NiCo_2_O_4_ nanomaterials’ catalytic activity; thus, Sample 1 was obtained in order to achieve optimized urea concentrations in NiCo_2_O_4_ nanomaterials for the development of non-enzymatic urea sensors. The nickel cobalt has shown redox behavior, indicating its good electrochemical properties with abundant active sites for catalyzing the urea oxidation. Generally, NiCo_2_O_4_ nanomaterials for urea detection can be characterized as follows by Equations (1) and (2) [31,32,33,34].

Ni(OH)_2_ + OH^−^ → NiOOH + H_2_O + e^−^ (1)

CO(NH_2_)_2_ + 6Ni(OOH) + H_2_O → N_2_ + 6Ni(OH)_2_ + CO_2_ + 6e^−^ (2)

**Figure 3:**
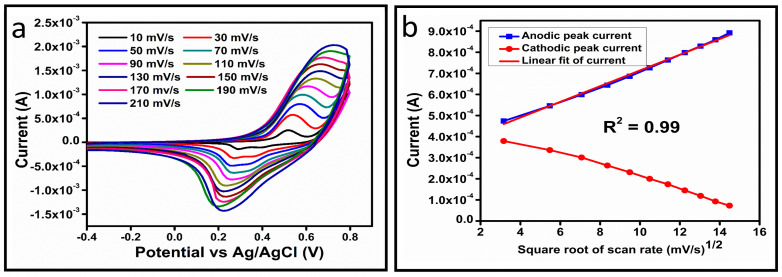
(**a**) CV curves of Sample 2 based on NiCo_2_O_4_ nanomaterial, at various scan rates in the presence of 0.1 mM urea prepared in 0.1 M NaOH, (**b**) Linear plots of anodic and cathodic peak currents.

**Figure 4:**
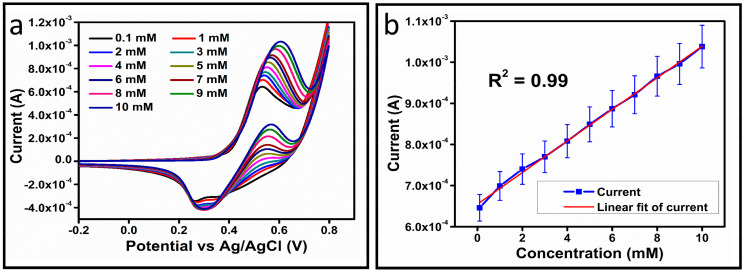
(**a**) CV curves of Sample 2 based on NiCo_2_O_4_ nanomaterial, at 50 mV/s in the presence of various urea concentrations prepared in 0.1 M NaOH, (**b**) Linear plot of oxidation peak current versus different urea concentrations.

**Figure 5:**
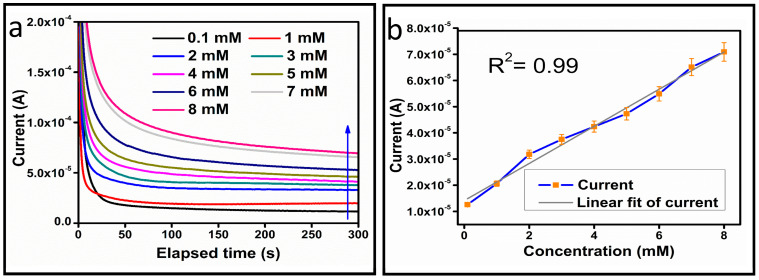
(**a**) chronoamperometry response of Sample 2 based on NiCo_2_O_4_ nanomaterial, at 0.6 V in the presence of various urea concentrations prepared in 0.1 M NaOH, (**b**) Linear plot of amperometeric generated current against various urea concentrations.

In these CV curves, it is evident that urea concentration has a significant impact on the catalytic activity of NiCo_2_O_4_ nanomaterial toward urea detection, thus optimizing the urea content for NiCo_2_O_4_ nanomaterial growth. In the current study, highly sensitive electrical signals were generated that could strengthen the development of an advanced urea enzyme-free sensing system. Our preliminary testing of urea detection has been followed by an electroanalytical characterization of Sample 1 in terms of charge transfer, stability, reproducibility, repeatability, and selectivity, as well as the practical application of the non-enzymatic urea sensor based on different real samples. Figure 3a illustrates the electrochemical behavior of Sample 1 using CV curves measured at different scan rates with 0.5 mM urea concentration. When the scan rate increased from 10–210 mV/s, the oxidation peak current increased linearly as shown in Figure 3a. During the increasing scan rate, the CV curves indicate both oxidation and reduction processes together, suggesting a reversible phenomenon. As shown in Figure 3b, both redox peak currents were linearly plotted against the square root of the scan rate, and r^2^: 0.99 is characteristic of a reliable and accurate analytical device. For the development of the electroanalytical method, the value of regression coefficient (R^2^) is suggesting the relative deviation in the method, and it is believed that a regression coefficient of 0.99 is considered as an standard value for the verification of accuracy of the proposed method. We have used the square root of the scan rate because our modified electrode showed the linear dependence of the peak current on the square root of scan rate. According to the scan investigation, modified glassy carbon electrodes have diffusion-controlled characteristics, providing a stable and reproducible electrical signal for the newly developed electrochemical urea sensor. The CV was employed at 50 mV/s in order to identify the linear range of urea detection by the newly developed non-enzymatic urea sensor (Sample 1), and the curves for the urea signal at different concentrations are presented in Figure 4a. A well-resolved oxidation peak with enhanced peak current has been observed with each increment of urea concentration. This confirms the suitability of Sample 1 for the alternative determination of urea from real samples. The linear plot in Figure 4b shows the peak current of urea oxidation against each concentration of urea as a means of understanding the analytical behavior of Sample 1. It is obvious from the linear plot that analytical aspects of Sample 1 were well supported by the regression coefficient of 0.99 and well linear fitting was achieved, as shown in Figure 4b. A linear range of 0.1 mM to 10 mM was found for urea sensing. Additionally, the low limit of detection (LOD) and limit of quantification (LOQ) were estimated based on the reported work [35]. Sample 1 demonstrated a LOD of 0.006 mM and a LOQ of 0.009 mM, demonstrating the promising nature of the presented non-enzymatic urea sensor and its potential application in real-life samples. Our CV results of linear were validated using the chronoamperometry technique at 0.6 V. Figure 5a shows the measured chronoamperometric signals for the proposed urea concentration. A chronoamperometric response for each concentration was found stable with a remarkably sensitive signal for a wide range of urea concentrations from 0.1 mM to 8 mM, conforming and strengthening the CV linear for urea detection. Using this chronoamperometric signal, Sample 1 was confirmed to have a high sensitivity to the non-enzymatic detection of urea. The sensitive urea sensing can be attributed to the role of urea concentration in making the surface of the NiCo_2_O_4_ nanomaterial highly conducive to urea detection. A plot of the linear fit of the generated amperometric current versus urea concentration is shown in Figure 5b. A regression coefficient of 0.99 indicates the NiCo_2_O_4_ nanomaterial could play a role in the quantification of urea from practical samples. In Table 1 [36,37,38,39,40,41], the proposed non-enzymatic urea sensor configuration is found to perform better than or as well as many of the recently published urea sensors/biosensors in terms of wide linear range, especially for the non-enzymatic urea sensors. The LOD of the presented is also superior to many of the reported works’ enzymatic and non-enzymatic sensors, as given in Table 1. The significant benefits of NiCo_2_O_4_ nanomaterial is that it is relatively low cost, easy to produce, and can be scaled up. This makes it an excellent alternative method for the detection of urea in biological fluids. The enzymatic urea biosensors have shown wide linear range [38,40]; however, the fabrication of urease based urea biosensors is very costly, involving the complicated immobilization of urea enzymes, and their storage issues limit their popularity. Currently, the development of non-enzymatic urea sensors has more advantage over urease based biosensors, owing to their low cost and long, simple storage life. Therefore, the presented non-enzymatic urea sensors based on NiCo_2_O_4_ nanostructures have a promising and potential capability to use as an alternative method to monitor the urea from real samples with high precision and accuracy. In comparison to our previous study [16], the performance of the presented non-enzymatic urea sensor can be differentiated in the following ways: using chronoamperometry, we evaluated the role of the variation of urea concentration while keeping the cobalt chloride fixed towards sensitive urea detection. The purpose of this study was to investigate the redox properties of NiCo_2_O_4_ in the presence of varying concentrations of urea for enzyme-free sensing applications of urea detection. In this study, three aspects differ from our previous work: (1) investigating the role of urea precursors in NiCo_2_O_4_, using chronoamperometry, a very sensitive technique; (2) the detection of urea within a wide linear range by Sample 2; and (3) the successful detection of urea in dairy products. Our main objective was to address the fundamental issue regarding the development of sensitive non-enzymatic sensors to quantify urea in clinical and dairy products by addressing the fundamental role of precursors. This is of critical importance to the development of sensitive non-enzymatic sensors.

**Figure 6:**
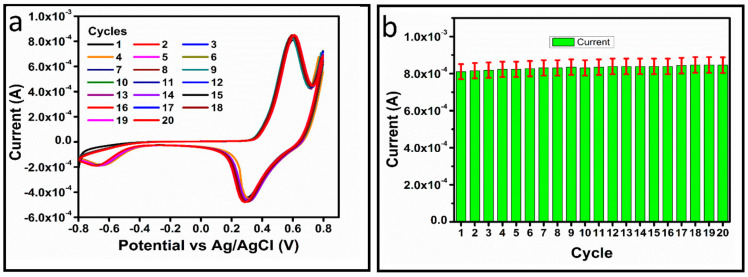
(**a**) CV curves of Sample 2 based on NiCo_2_O_4_ nanomaterial for the monitoring of stability and repeatability, at 50 mV/s in the presence of 0.1 mM urea concentration prepared in 0.1 M NaOH, (**b**) illustration of peak current in bar graph for the statistical error for the demonstration of stability and repeatability of material.

Furthermore, the electrochemical properties of the proposed sensor are highly sensitive, selective, and stable when compared with previous studies. The selective qualities of as-prepared Sample 1 were also determined by competing agents during urea detection and various interfering species, including ascorbic acid, uric acid, glucose, magnesium ions, and potassium ions, were sequentially added in the presence of urea. Despite successive additions of interfering substances, the peak current was not changed significantly when equal concentrations of urea and other species (0.1 mM) were used, confirming the highly selective nature of Sample 1 towards urea. The selectivity of non-enzymatic sensors is mainly governed by the nature of electrocatalytic material; hence, electrocatalytic features of materials have already been tested and studied for only a specific type of application, as reported elsewhere. This is the reason intensive studies about the development of highly electrocatalytic materials have been carried out, owing to their high selectivity towards a particular application. We examined the repeatability and stability of Sample 1 in the presence of 0.1 mM of urea by examining the number of CV cycles, as shown in Figure 6a. As shown by the repeatability of CV cycles, there was no potential shift, and the peak current of urea oxidation indicated the material could be used for long-term quantification of urea. A repeatable CV cycle for the illustration of change in the peak current was performed, and the standard deviation was less than 3% (Figure 6b), which confirms the high repeatability of the non-enzymatic urea sensor for the detection of urea under alkaline conditions.

**Figure 7:**
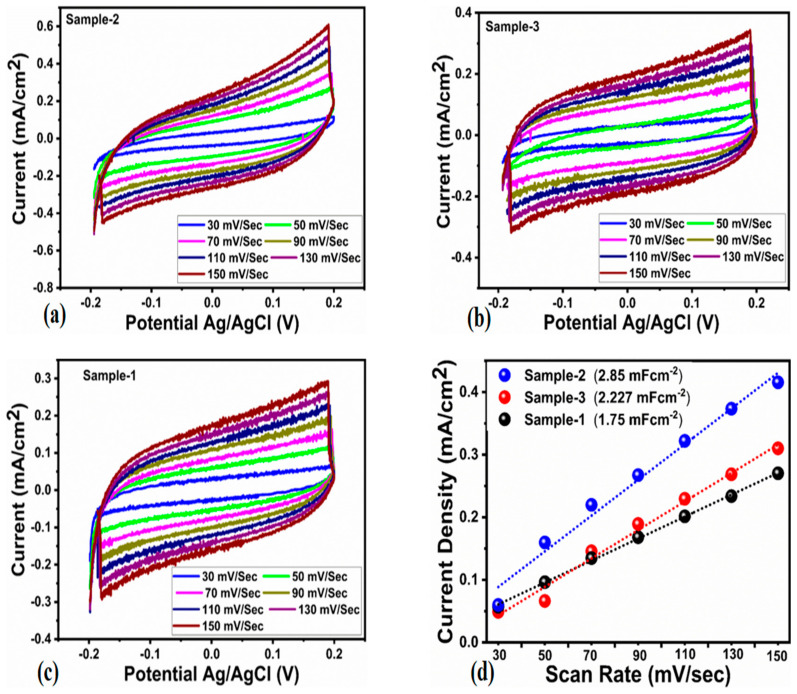
(**a**–**c**) Cyclic voltammetry measured for Samples 2 and 3 and Sample 1 in 0.5 mM urea solution at different scan rates. (**d**) Linear plots of anodic and cathodic current density difference for each scan rate for the quantification of (ECSA).

The active surface area of different NiCo_2_O_4_ nanomaterials were also estimated through CV curves measured at various scan rates, as shown in Figure 7. The corresponding CV curves for Samples 1–3 at different scan rates were recorded in 0.5 mM urea and are shown in Figure 7a–c, respectively. It could be seen that the behavior of curves for three samples was found non-Faradic, and they have been used for the calculation of electrochemical active surface area (ECSA) through the difference of anodic and cathodic current density for each scan rate, as shown in Figure 7d. The linear plot shown in Figure 7d indicates the corresponding value of ECSA for Samples 1–3 as 1.75 mFcm^−2^, 2.225 mFcm^−2^, and 2.85 mFcm^−2^, respectively. The high active surface area for Sample 2 strongly supports its enhanced electrocatalytic performance towards the sensitive and selective detection of urea.

### 3.3. Real Sample Analytical Application of Proposed Non-Enzymatic Urea Sensor

The practicality of this non-enzymatic urea sensor was demonstrated using milk and urine samples prepared for analysis by the non-enzymatic urea sensor using a 1:30 volume ratio. As a result of repeating the analysis of the real sample three times, Table 2 provides the reported results. The urea sensor shows accurate determination of urea from milk and urine samples, proving the reliability and practicality of the proposed non-enzymatic urea sensor. The low values of relative standard deviation (RSD) of less than 1% verify the promising urea determination from real samples of milk and urine. The RSD (%) were estimated using (standard deviation/average of quantified urea data by 3 repeatable cycle experiments of urea sensing) ×100%.

## 4. Conclusions

In this study, we investigated the effect of urea concentration on the non-enzymatic sensing activity of NiCo_2_O_4_ nanomaterials in alkaline media. The hydrothermal method was used to prepare three samples of NiCo_2_O_4_ nanomaterials with different concentrations of urea. XRD and SEM techniques were used to investigate the structure and chemical composition of the sample. Various electrochemical methods were employed to evaluate the performance of NiCo_2_O_4_ nanomaterials, including cyclic voltage measurement and chronoamperometry. Based on CV analysis, the linear range for Sample 1 based on NiCo_2_O_4_ nanomaterial was found to be 0.1 mM to 10 mM, and for the chronoamperometry signal to be 0.1 mM to 8 mM. Additionally, an estimate of the LOD and LOQ of 0.006 mM and 0.009 mM was made. It was also found that the stability, repeatability, and selectivity studies were satisfactory. The practical component of the designed urea sensor was assessed by evaluating milk and urine samples, which demonstrated the sensor’s suitability for practical applications in urea regulation and monitoring.

## Figures and Tables

**Table 1:** Performance evaluation comparative analysis of NiCo_2_O_4_ with recently published urea biosensors/sensors.

Sensing Material	Linear Range (mM)	Limit of Detection (LOD) (μM)	Method of Detection	Reference
NiO–MoO_3_	0.2–1	0.86	Non-enzymatic	[36]
NiCo_2_O_4_	0.01–5	1	Non-enzymatic	[16]
NiS/GO/MGCE	0.1–1.0	3.79	Non-enzymatic sensor	[37]
Urease/ZrO_2_ thin film/Au	0.8–16.6	442	Enzymatic	[38]
Ni(OH)_2_/Mn_3_O_4_/ rGO/PANi	0.03–3.3	16.3	Non-enzymatic	[39]
(Ni-MOF) nanobelts	0.01–7.0	2.23	Non-enzymatic	[40]
nano-PANI:PSS	0.2–0.9	919.3	Enzymatic	[41]
NiCo_2_O_4_ nanorods	0.1–10 0.1–8	6	Non-enzymatic (CV) (chronoamperometry	This work

**Table 2:** Percent recovery and direct quantification of urea from milk, and urine real sample analysis using presented non-enzymatic urea sensors based on urea derived Co_2_O_4_ nanomaterial.

Sample	Added (mM)	Found (mM)	(%) Recovery	(%) RSD
Urine	-	2.3	-	
-	0.5	2.81 ± 0.002	100.35	0.42
-	1	3.32 ± 0.003	100	0.37
Milk 1	-	1.95	-	
-	1	2.94 ± 0.005	99	0.30
-	1.5	3.46 ± 0.007	100	0.34
Milk 2		2.4	-	
-	1.5	3.91 ± 0.004	100.25	0.27
-	2.5	4.89 ± 0.002	99.79	0.30

## Data Availability

The data presented in this study are available on request from the corresponding author.

## References

[1] Jonker J., Kohn R., Erdman R. (1998). Using milk urea nitrogen to predict nitrogen excretion and utilization efficiency in lactating dairy cows. J. Dairy Sci..

[2] Roy B., Brahma B., Ghosh S., Pankaj P., Mandal G. (2011). Evaluation of milk urea concentration as useful indicator for dairy herd management: A review. Asian J. Anim. Vet. Adv..

[3] Francis P.S., Lewis S.W., Lim K.F. (2002). Analytical methodology for the determination of urea: Current practice and future trends. TrAC Trends Anal. Chem..

[4] Bisht V., Takashima W., Kaneto K. (2005). An amperometric urea biosensor based on covalent immobilization of urease onto an electrochemically prepared copolymer poly (N-3-aminopropyl pyrrole-co-pyrrole) film. Biomaterials.

[5] Deng H.-H., Hong G.-L., Lin F.-L., Liu A.-L., Xia X.-H., Chen W. (2016). Colorimetric detection of urea, urease, and urease inhibitor based on the peroxidase-like activity of gold nanoparticles. Anal. Chim. Acta.

[6] Jha S.N., Jaiswal P., Borah A., Gautam A.K., Srivastava N. (2015). Detection and quantification of urea in milk using attenuated total reflectance-Fourier transform infrared spectroscopy. Food Bioprocess Technol..

[7] Childers C.L., Green S.R., Dawson N.J., Storey K.B. (2016). Native denaturation differential scanning fluorimetry: Determining the effect of urea using a quantitative real-time thermocycler. Anal. Biochem..

[8] Xie W.-Q., Yu K.-X., Gong Y.-X. (2019). Rapid and quantitative determination of urea in milk by reaction headspace gas chromatography. Microchem. J..

[9] Boggs B.K., King R.L., Botte G.G. (2009). Urea electrolysis: Direct hydrogen production from urine. Chem. Commun..

[10] Abernethy G., Higgs K. (2013). Rapid detection of economic adulterants in fresh milk by liquid chromatography–tandem mass spectrometry. J. Chromatogr. A.

[11] Ma W.-J., Luo C.-H., Lin J.-L., Chou S.-H., Chen P.-H., Syu M.-J., Kuo S.-H., Lai S.-C. (2016). A portable low-power acquisition system with a urease bioelectrochemical sensor for potentiometric detection of urea concentrations. Sensors.

[12] Nie F., Wang N., Xu P., Zheng J. (2017). Determination of urea in milk based on N-bromosuccinimide–dichlorofluorescein postchemiluminescence method. J. Food Drug Anal..

[13] Chang A.S., Tahira A., Chang F., Solangi A.G., Bhatti M.A., Vigolo B., Nafady A., Ibupoto Z.H. (2023). Highly Heterogeneous Morphology of Cobalt Oxide Nanostructures for the Development of Sensitive and Selective Ascorbic Acid Non-Enzymatic Sensor. Biosensors.

[14] Chang A.S., Tahira A., Solangi Z.A., Solangi A.G., Ibupoto M.H., Chang F., Medany S.S., Nafady A., Kasry A., Willander M. (2022). Pd-Co3O4-based nanostructures for the development of enzyme-free glucose sensor. Bull. Mater. Sci..

[15] Ansari S., Fouad H., Shin H.-S., Ansari Z. (2015). Electrochemical enzyme-less urea sensor based on nano-tin oxide synthesized by hydrothermal technique. Chem. Biol. Interact..

[16] Amin S., Tahira A., Solangi A., Beni V., Morante J., Liu X., Falhman M., Mazzaro R., Ibupoto Z.H., Vomiero A. (2019). A practical non-enzymatic urea sensor based on NiCo_2_O_4_ nanoneedles. RSC Adv..

[17] Nguyen N.S., Das G., Yoon H.H. (2016). Nickel/cobalt oxide-decorated 3D graphene nanocomposite electrode for enhanced electrochemical detection of urea. Biosens. Bioelectron..

[18] Mondal S., Sangaranarayanan M. (2013). A novel non-enzymatic sensor for urea using a polypyrrole-coated platinum electrode. Sens. Actuators B Chem..

[19] Ding R., Qi L., Jia M., Wang H. (2013). Porous NiCo_2_O_4_ nanostructures as bi-functional electrocatalysts for CH3OH oxidation reaction and H_2_O_2_ reduction reaction. Electrochim. Acta.

[20] Hassanpoor S., Aghely F. (2020). Hierarchically self-assembled NiCo_2_O_4_ nanopins as a high-performance supercapacitor cathodic material: A morphology controlled study. RSC Adv..

[21] Yu Z., Li H., Zhang X., Liu N., Tan W., Zhang X., Zhang L. (2016). Facile synthesis of NiCo_2_O_4_@ Polyaniline core–shell nanocomposite for sensitive determination of glucose. Biosens. Bioelectron..

[22] Zhan J., Cai M., Zhang C., Wang C. (2015). Synthesis of mesoporous NiCo2O4 fibers and their electrocatalytic activity on direct oxidation of ethanol in alkaline media. Electrochim. Acta.

[23] Yu H., Jin J., Jian X., Wang Y., Qi G.C. (2013). Preparation of cobalt oxide nanoclusters/overoxidized polypyrrole composite film modified electrode and its application in nonenzymatic glucose sensing. Electroanalysis.

[24] Pasta M., La Mantia F., Cui Y. (2010). Mechanism of glucose electrochemical oxidation on gold surface. Electrochim. Acta.

[25] Saraf M., Natarajan K., Mobin S.M. (2017). Multifunctional porous NiCo_2_O_4_ nanorods: Sensitive enzymeless glucose detection and supercapacitor properties with impedance spectroscopic investigations. New J. Chem..

[26] Amin B.G., Masud J., Nath M. (2019). A non-enzymatic glucose sensor based on a CoNi_2_Se_4_/rGO nanocomposite with ultrahigh sensitivity at low working potential. J. Mater. Chem. B.

[27] Li H., Zhang L., Mao Y., Wen C., Zhao P. (2019). A simple electrochemical route to access amorphous Co-Ni hydroxide for non-enzymatic glucose sensing. Nanoscale Res. Lett..

[28] Solangi A.G., Pirzada T., Shah A.A., Halepoto I.A., Chang A.S., Solangi Z.A., Solangi M.Y., Aftab U., Tonezzer M., Tahira A. (2022). Phytochemicals of mustard (Brassica Campestris) leaves tuned the nickel-cobalt bimetallic oxide properties for enzyme-free sensing of glucose. J. Chin. Chem. Soc..

[29] Li J., Chen Z., Li Y. (2011). A strategy for constructing sensitive and renewable molecularly imprinted electrochemical sensors for melamine detection. Anal. Chim. Acta.

[30] Kumar T.V., Sundramoorthy A.K. (2018). Non-enzymatic electrochemical detection of urea on silver nanoparticles anchored nitrogen-doped single-walled carbon nanotube modified electrode. J. Electrochem. Soc..

[31] Vedharathinam V., Botte G.G. (2012). Understanding the electro-catalytic oxidation mechanism of urea on nickel electrodes in alkaline medium. Electrochim. Acta.

[32] Daramola D.A., Singh D., Botte G.G. (2010). Dissociation rates of urea in the presence of NiOOH catalyst: A DFT analysis. J. Phys. Chem. A.

[33] Vedharathinam V., Botte G.G. (2013). Direct evidence of the mechanism for the electro-oxidation of urea on Ni(OH)_2_ catalyst in alkaline medium. Electrochim. Acta.

[34] Guo F., Ye K., Du M., Huang X., Cheng K., Wang G., Cao D. (2016). Electrochemical impedance analysis of urea electro-oxidation mechanism on nickel catalyst in alkaline medium. Electrochim. Acta.

[35] Chang A.S., Tahira A., Chang F., Memon N.N., Nafady A., Kasry A., Ibupoto Z.H. (2021). Silky Co_3_O_4_ nanostructures for the selective and sensitive enzyme free sensing of uric acid. RSC Adv..

[36] Salarizadeh N., Habibi-Rezaei M., Zargar S.J. (2022). NiO–MoO_3_ nanocomposite: A sensitive non-enzymatic sensor for glucose and urea monitoring. Mater. Chem. Phys..

[37] Naik T.S.K., Saravanan S., Saravana K.S., Pratiush U., Ramamurthy P.C. (2020). A non-enzymatic urea sensor based on the nickel sulfide/graphene oxide modified glassy carbon electrode. Mater. Chem. Phys..

[38] Sumana G., Das M., Srivastava S., Malhotra B.D. (2010). A novel urea biosensor based on zirconia. Thin Solid Film..

[39] Nia S.M., Kheiri F., Jannatdoust E., Sirousazar M., Chianeh V.A., Kheiri G. (2021). A Highly Sensitive Non-Enzymatic Urea Sensor Based on Ni (OH)_2_/Mn_3_O_4_/rGO/PANi Nanocomposites Using Screen-Printed Electrodes. J. Electrochem. Soc..

[40] Bao C., Niu Q., Chen Z.-A., Cao X., Wang H., Lu W. (2019). Ultrathin nickel-metal–organic framework nanobelt based electrochemical sensor for the determination of urea in human body fluids. RSC Adv..

[41] Soni A., Surana R.K., Jha S.K. (2018). Smartphone based optical biosensor for the detection of urea in saliva. Sens. Actuators B Chem..

